# Long-term Interactions of Circulating Neutrophils with Titanium Implants, the Role of Platelets in Regulation of Leukocyte Function

**DOI:** 10.3390/ijms221810060

**Published:** 2021-09-17

**Authors:** Joanna Zdziennicka, Andrzej Junkuszew, Michał Latalski, Michał Świeca, Joanna Wessely-Szponder

**Affiliations:** 1 Sub-Department of Pathophysiology, Department of Preclinical Veterinary Sciences, Faculty of Veterinary Medicine, University of Life Sciences, 20-033 Lublin, Poland; joanna.michalska15@gmail.com; 2 Faculty of Animal Sciences and Bioeconomy, Institute of Animal Breeding and Biodiversity Conservation, University of Life Sciences, 20-950 Lublin, Poland; andrzej.junkuszew@up.lublin.pl; 3 Children Orthopaedic Department, Medical University of Lublin, 20-093 Lublin, Poland; michallatalski@umlub.pl; 4 Department of Biochemistry and Food Chemistry, University of Life Sciences, 20-704 Lublin, Poland; michal.swieca@up.lublin.pl

**Keywords:** antimicrobial peptides, platelet-rich plasma, titanium implant, neutrophils, pentoxifylline

## Abstract

Despite the fact that different biomaterials are widely used in many biomedical applications, they can still cause side effects. Therefore, our aim was to assess neutrophil activity during the inflammatory phase of the repair process and long-term interactions between circulating neutrophils and Titanium (Ti) implants. Additionally, neutrophil in vitro response after stimulation by the extract of antimicrobial peptides (AMP extract), pentoxifylline (PTX) and some platelet-rich (L-PRP and PURE PRP) and platelet-poor (PPP) concentrates were tested. The study was conducted on eight sheep after Ti implant insertion into the tibia and revealed that the Ti implant did not cause any side effects during the course of experiment. After addition of L-PRP into neutrophils, culture activity of these cells significantly increased (*p* < 0.01), whereas treatment with AMP extract, PURE PRP, PPP or PTX caused decrease in neutrophil enzymatic response (on the basis of elastase, myeloperoxidase and alkaline phosphatase release) and free radical generation. These effects were observed in neutrophils isolated during the inflammatory phase as well as 4 and 10 months after implantation. Obtained results will be useful in regulation of inflammatory response during implantation of biomaterial and create possibility to modulate the cells response towards pro- or anti-inflammatory to reduce host tissue damage.

## 1. Introduction

Biomaterials are essential for a variety of healthcare applications and favored the great improvements in tissue engineering, medical implants applications, drug delivery, and immunotherapy [1]. In regenerative medicine, for evaluation of the bone substitute behavior at the early stages of bone repair, combinations of biomaterials, such as autologous human bone, porcine bone or a mixture of both were used in human clinical study [2]. However, the common property of biomaterials is the induction of adverse immune reactions resulting in excessive inflammatory response, impaired healing process, fibrotic encapsulation, and tissue destruction [1]. These adverse effects of bone implants are widely recognized as a significant cause of aseptic implant failure. Within these effects, osteolysis around the implant is caused by the activation of different cells and secretion of pro-inflammatory cytokines. This deleterious response could be evoked by particulate wear and corrosion debris, organo–metallic complexes, metal salt/oxides and free metal ions released from implant [3,4,5]. Titanium (Ti) is the most common metal used in orthopedics and dentistry. Despite the fact that this metal is highly resistant to corrosion, Ti ions might slowly diffuse into surrounding tissue and might be transported into plasma and interact with circulating blood cells. It was confirmed that particles formed by Ti ions can act as secondary stimuli to activate and release pro-inflammatory cytokines from human macrophages; furthermore, after exposition to Ti particles macrophages are able to cause neutrophil recruitment [6]. Due to the increasing use of titanium implants in clinical practice, it should be borne in mind that complications rate will also increase. Therefore, understanding the mechanisms responsible for the formation of aseptic implant rejection, especially the role of immune cells in this process is of growing interest.

Polymorphonuclear neutrophils (PMNs) appeared to be a non-homogenous population. Apart from their microbicidal role in the first-line defense they have been recently recognized as effector cells responsible for regulation of inflammation [7]. On one hand they can cause inflammatory injury of different tissues and organs and are not indicated for some applications [8]; in particular, excessive enzyme release from neutrophils and reactive oxygen species (ROS) generation can cause host tissue injury [9]. On the other hand, the “regulatory” or “suppressor” neutrophils subtype can express some Toll-like receptors (TLR) and can induce an anti-inflammatory response, either by interacting directly with other cells or by secreting factors that induce polarization of other cell types [7]. Therefore, PMNs greatly impact immune mechanisms of the host, thus they significantly contribute to immune modulation, tissue repair and local inflammation which is necessary in chronic wound repair or bone healing. For this reason, we evaluated some products as potential modifiers of neutrophil response. Autologous neutrophil extract was previously recognized as the source of antimicrobial peptides [10] and considered as the factor able to decrease the excessive response of leukocytes [11,12]. Another potential modulator of neutrophil activity, Pentoxifylline (PTX) is a competitive non-selective phosphodiesterase inhibitor, which acts as an anti-inflammatory, enhances microcirculation, blood flow and tissue oxygenation. PTX decreases superoxide generation and neutrophil degranulation [13]. It also stimulates bone formation and increases systemic bone mass in animal models; thus, it could be considered in some approaches to improve osseointegration [14].

Platelet-rich plasma (PRP) and other platelet concentrates have been used in regenerative medicine for long time. In the light of fact that platelets can interact with PMNs and are considered as regulators of inflammation through TLR expression, it seemed to be interesting to evaluate the co-culture of both cell populations [9,15]. In veterinary medicine the use of PRP started from the treatment of ligament and tendons injuries in horses with osteoarthrosis, and currently it is used for many surgical procedures in human medicine and in animals [16]. Some authors found that the combination of neutrophils and activated platelets could have more positive than detrimental effects on tissue repair, especially in the regenerative phase in the inflammatory process [17]. Generally, it was emphasized that the effectiveness of PRP is attributed to an optimal balance between the anabolic effect of growth factors and the catabolic effect of cytokines as well as a proper platelets/leukocytes ratio, depending on the potential application [18]. To date there are no clear regulations of the preparation and the content of PRP. The composition of this blood-derived product varies greatly in platelets’, white blood cells’ (WBC) and red blood cells’ (RBC) contents. Therefore, PRP has to be considered and evaluated as a few different products, such as leukocyte-rich PRP (LR-PRP), leukocyte-poor PRP (LP-PRP), clinical PRP (C-PRP) amongst others. Additionally, the PRP by-product named platelet poor plasma fraction (PPP) rich in plasma proteins (fibrinogen, fibronectin, and thrombin) could be added to these products and used in some applications, as it promotes wound healing, and accelerates cell migration and proliferation of fibroblasts [8,19]. Previously, leukocyte- and platelet-rich fibrin (L-PRF) was successfully applied in treatment of periodontal disorders to enhance tissue regeneration by stimulation of specific cell anabolic activities. This compound appeared to be a low-cost, easy to use and effective platelet-derived concentrate [20].

Platelet-related products including PRP, PPP and platelet-rich fibrin are widely used to promote wound healing and tissue regeneration. Thus, it is important from the clinical point of view to study the differences between these products in terms of composition and their biologic effects [19]. In our study based on the platelet and leukocyte content, platelet-related products have been classified as PURE-PRP containing a few or no leukocytes, LR-PRP (leukocyte-rich PRP) that contains more leukocytes and PPP with a low concentration of platelets [21].

In-depth understanding of the host/biomaterial interactions is strongly needed to develop procedures to overcome side effects during the application of biomaterials, which are still an important challenge in the regenerative medicine [1]. A part of this knowledge is neutrophil interactions with Ti [22]. Previously, some authors stated that neutrophils as the leading participants of the inflammatory phase have the potential to modulate inflammatory response to Ti implantation [23]. Kumazawa, in turn [6], examined the effect of Ti on the function and morphology of the human neutrophils and tested their cytotoxicity and biocompatibility in vitro and in vivo. We evaluated the potential of ovine PMNs as regulatory cells both in the inflammatory phase of repair process and on long-term interactions with Ti implantation. We also assessed some products as potential regulators of neutrophil inflammatory response. In this study AMP extract, PTX and different platelet-rich or platelet-poor concentrates were evaluated in terms of their interactions with circulating neutrophils after Ti implant insertion. Our aim was to assess PMNs response to overall conditions involved in implantation of biomaterial into the tibia, including anesthesia, implantation of a Ti implant and post-operative management. In the subsequent part of the experiment the long-term interactions between Ti implant and circulating neutrophils were evaluated. To our knowledge this is the first study describing these interactions on an ovine model of the Ti implant insertion.

## 2. Results

### 2.1. Clinical Results

Over the course of the experiment, no adverse effects at the implantation site were observed for up to 10 months after the implantation of Ti implants. All sheep were examined for possible implantation failure; clinical examinations did not reveal any pathological signs and radiological findings confirmed proper implantation. Hematological and acute phase proteins (APP) values were within the normal range (Table 1).

### 2.2. Activity of Neutrophils Isolated during the Inflammatory Phase of Repair after Implantation

**Figure 1:**
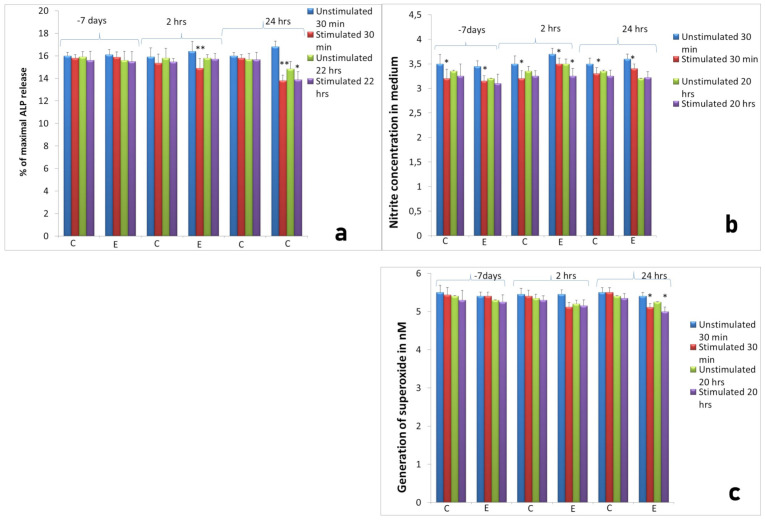
Activity of neutrophils isolated 7 days before, 2 h and 24 h after implantation (experimental: 8 sheep) compared to control groups of neutrophils isolated at the same time points from animals from control groups (control: 4 sheep). C: control group, E: experimental group. The neutrophil response to Ti implant insertion, with or without stimulation with AMP extract; (**a**) ALP release from neutrophils, (**b**) NO generation release, (**c**) superoxide generation in the inflammatory phase of the repair process assessed at two measurements, after 30 min and 22 h incubation at 37 °C and 5% CO_2_. Data present mean values ± SE of at least three replicates for each bar. * *p* < 0.05 compared to cultures of neutrophils from the control group at the same time points. ** *p* < 0.01.

We did not observe any significant differences between the activity of neutrophils isolated 2 h and 24 h after implantation compared to neutrophils isolated at the same time points from control animals (Figure 1).

AMP extract evoked insignificant decrease in the release of elastase and MPO from primary neutrophil granules at all time points, 7 days before implantation, 2 h and 24 h after implantation in both measurements, after 30 min and 20 h incubation at 37 °C and 5% CO_2_ (Figure not shown). Significant (*p* < 0.01) decrease in enzymatic activity was noted after measurement of the release of ALP from secondary neutrophil granules at both time points after surgery (Figure 1a). Decrease in nitrite concentration (as an indicator of NO generation) was significant after 30 min culture in all groups and at the second time point after 20 h incubation (Figure 1b). Similar to the NO generation, superoxide generation significantly diminished in neutrophil cultures isolated from experimental sheep 24 h after implantation in cultures incubated for 30 min and for 20 h (Figure 1c). Together, these results confirmed the inhibitory effect of autologous AMP extract on neutrophil secretory activity.

**Figure 2:**
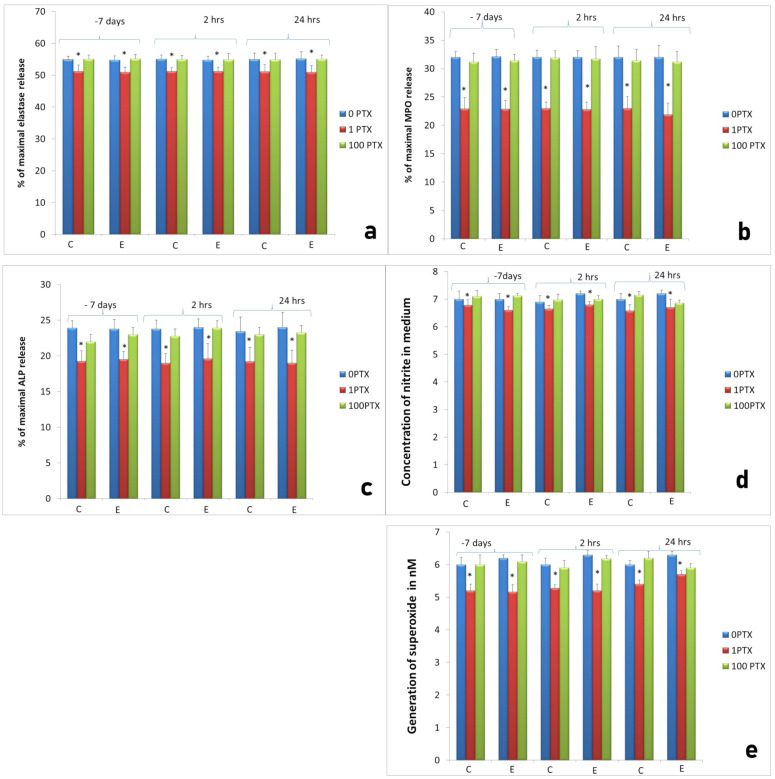
Activity of neutrophils isolated 7 days before, 2 h and 24 h after implantation (experimental: 8 sheep) compared to control groups of neutrophils isolated at the same time points from animals from control groups (control: 4 sheep). C: control group, E: experimental group. The response to implantation and treatment with two concentrations of PTX (1 and 100 µg/mL); (**a**) elastase release from neutrophils, (**b**) MPO release, (**c**) ALP release, (**d**) NO generation, (**e**) superoxide generation in the inflammatory phase of the repair process. Data present mean values ± SE of at least three replicates for each bar. * *p* < 0.05 compared to cultures of neutrophils without stimulation (0 µg/mL) at the same time points.

We demonstrated that the effect of PTX on neutrophil response was concentration-dependent. After stimulation with 1 µg/mL the significant decrease in neutrophils’ enzymatic response was noted. In cultures of neutrophils isolated from sheep two hours after implantation, elastase release was 51.2 ± 0.2% of maximal release (*p* < 0.05) compared to unstimulated culture of neutrophils from this measurement (54.8 ± 0.3%). Similar effects were seen in the release of other studied neutrophil products, namely MPO, ALP, NO and superoxide. On the other hand, the changes in neutrophil activity caused by PTX at a concentration of 100 µg/mL were insignificant compared to unstimulated cultures (Figure 2).

**Figure 3:**
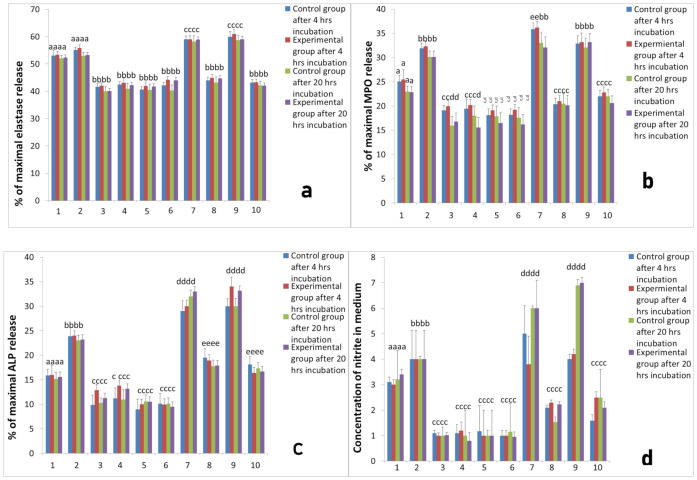

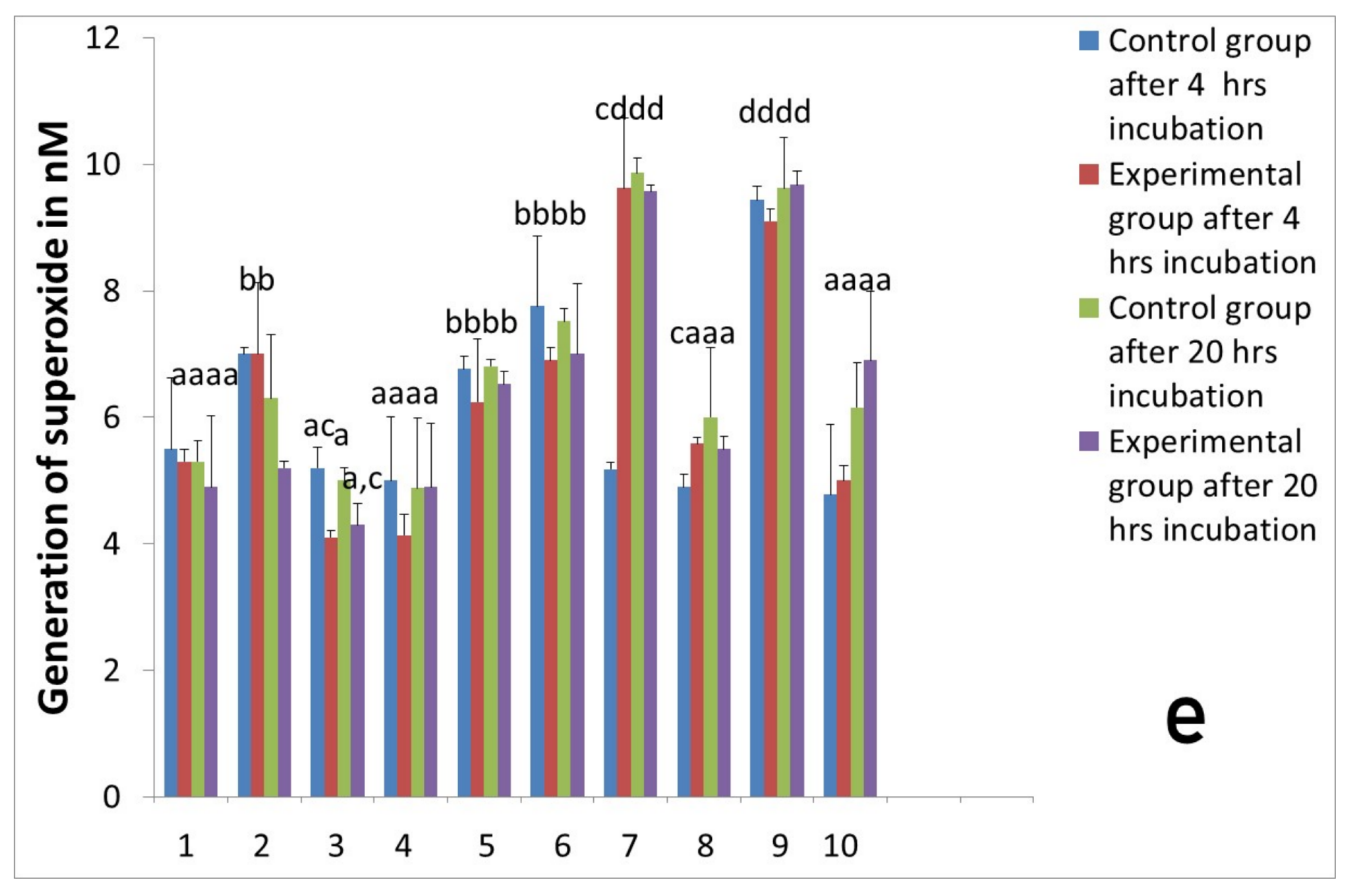
The response of neutrophils isolated before implantation with different PRP-derived products with or without additional stimulation with LPS. Legends: cultures were incubated for 4 h or for 20 h at 37 °C and 5% CO_2_. Data present mean values ± SE of at least three replicates for each bar. The neutrophil response was assessed with respect to: (**a**) elastase release from neutrophils, (**b**) MPO release, (**c**) ALP release, (**d**) NO generation, (**e**) superoxide generation. Values marked with different letters differed significantly (*p* < 0.05). Legends: 1—neutrophil without stimulation, 2—after stimulation LPS, 3—after stimulation PPP from L-PRP, 4—after stimulation PPP from PURE PRP, 5—after stimulation PPP from L-PRP with LPS, 6—after stimulation PPP from PURE PRP with LPS, 7—after stimulation L-PRP, 8—after stimulation PURE PRP, 9—after stimulation L—PRP with LPS, 10—after stimulation PURE PRP with LPS.

**Figure 4:**
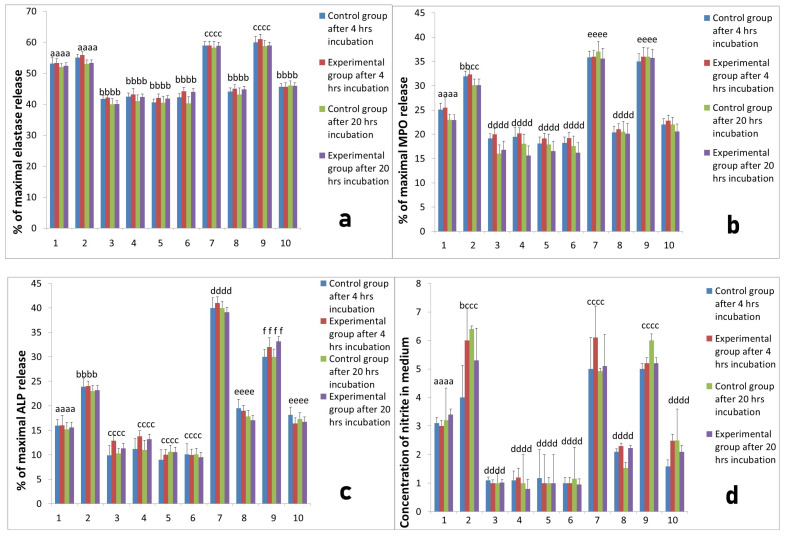

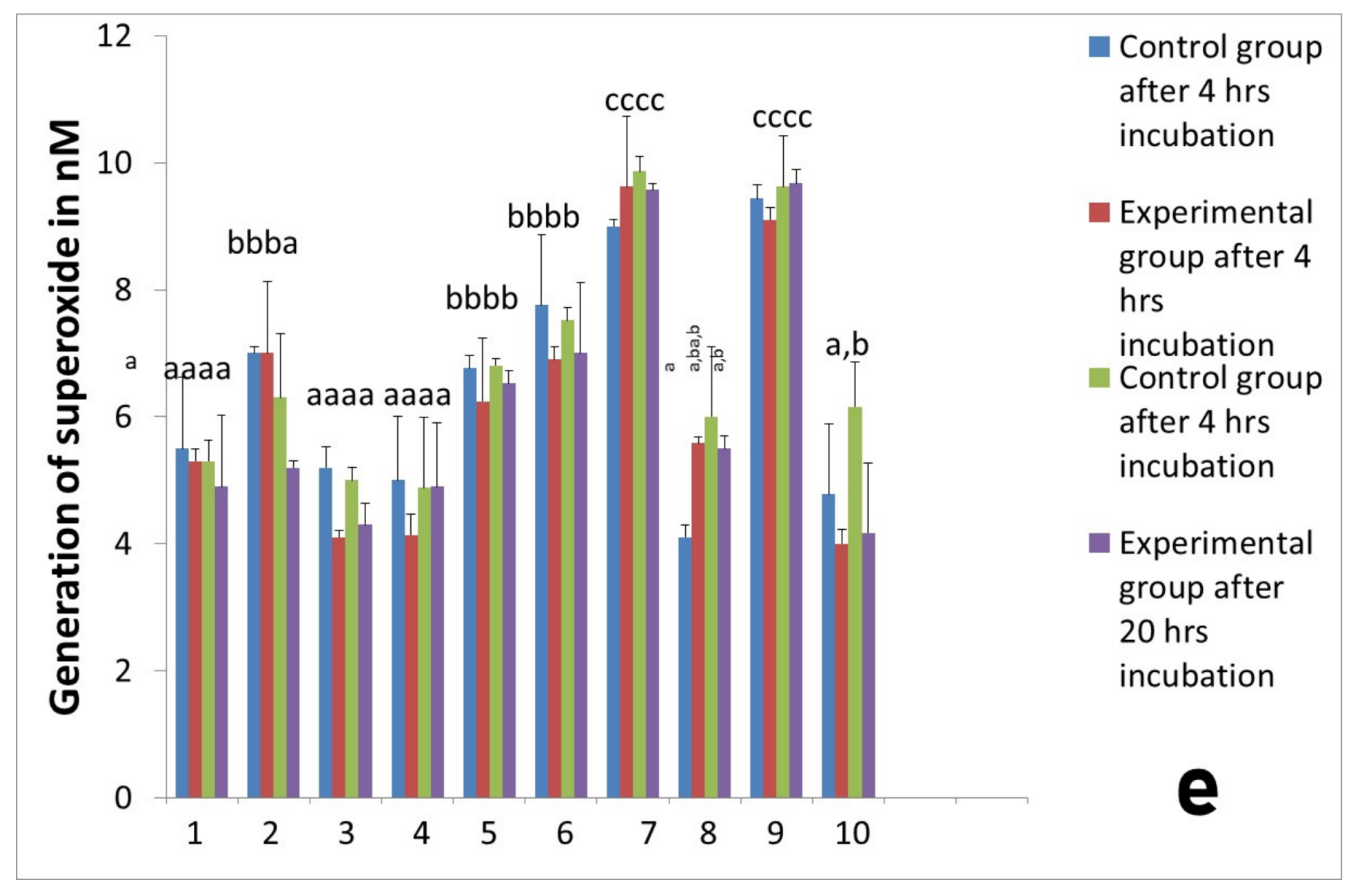
The response of neutrophils isolated 2 h after implantation with different PRP-derived products with or without additional stimulation with LPS. Cultures were incubated for 4 h or for 20 h at 37 °C and 5% CO_2._ Data present mean values ± SE of at least three replicates for each bar. The neutrophil response was assessed with respect to: (**a**) elastase release from neutrophils, (**b**) MPO release, (**c**) ALP release, (**d**) NO generation, (**e**) superoxide generation. Values marked with different letters differed significantly (*p* < 0.05). Legends: 1—neutrophil without stimulation, 2— after stimulation LPS, 3—after stimulation PPP from L-PRP, 4—after stimulation PPP from PURE PRP, 5—after stimulation PPP from L-PRP with LPS, 6—after stimulation PPP from PURE PRP with LPS, 7—after stimulation L-PRP, 8—after stimulation PURE PRP, 9—after stimulation L-PRP with LPS, 10—after stimulation PURE PRP with LPS.

**Figure 5:**
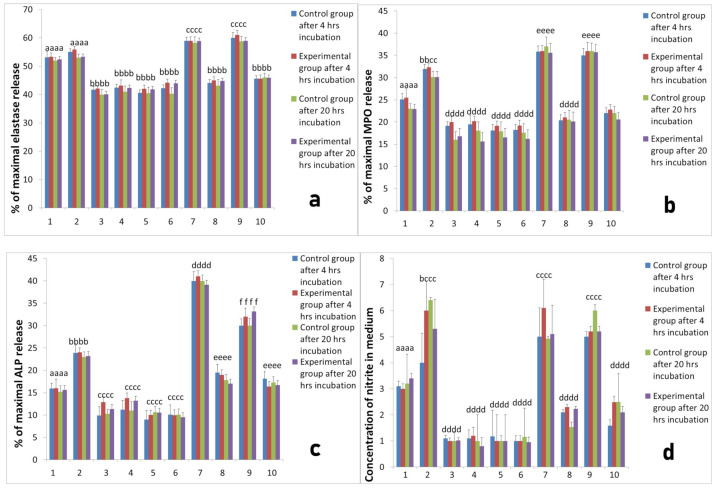

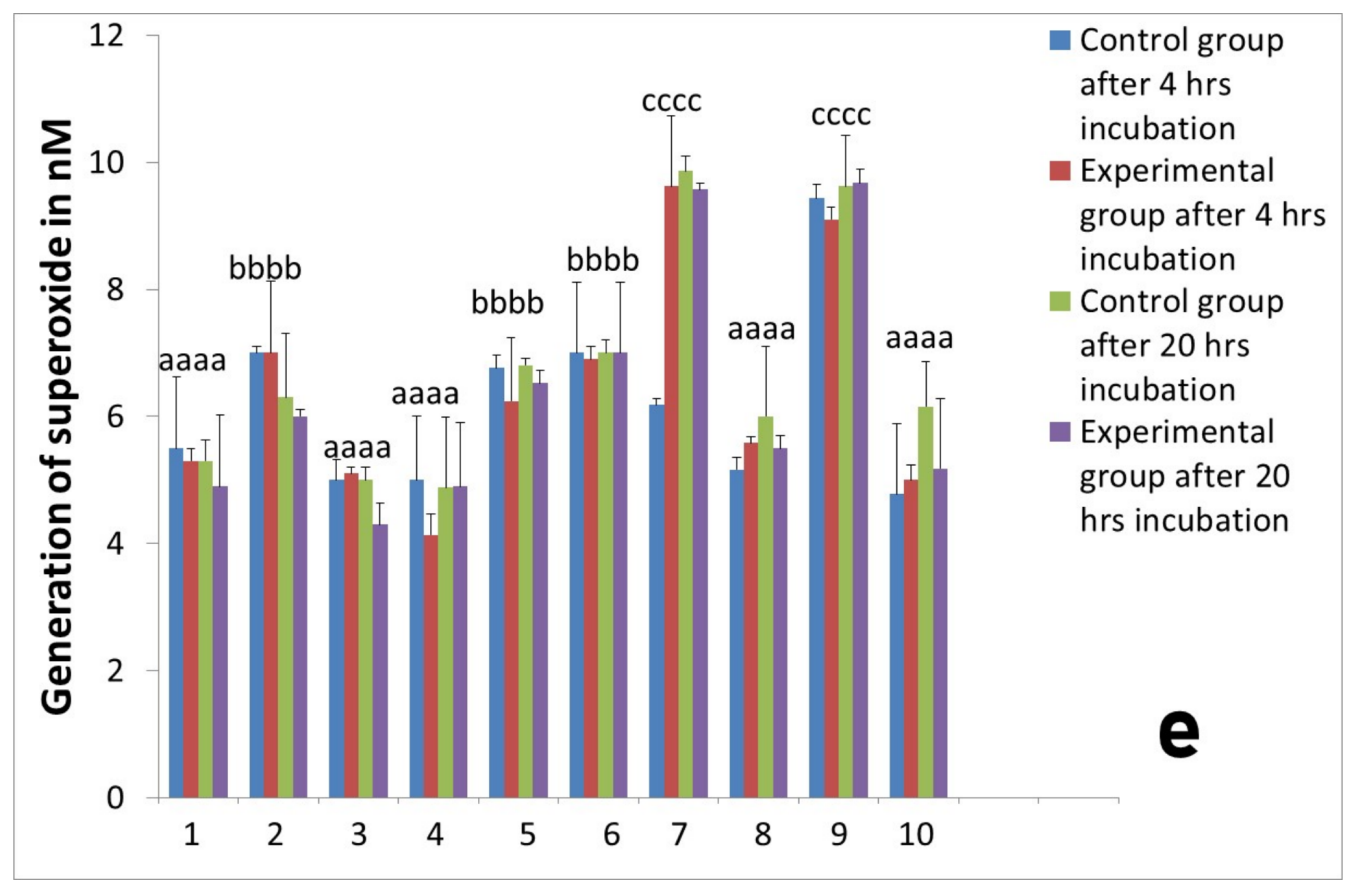
The response of neutrophils isolated 24 h after implantation with different PRP-derived products with or without additional stimulation with LPS. Cultures were incubated for 4 h or for 20 h at 37 °C and 5% CO_2._ Data present mean values ± SE of at least three replicates for each bar. The neutrophil response was assessed with respect to: (**a**) elastase release from neutrophils, (**b**) MPO release, (**c**) ALP release, (**d**) NO generation, (**e**) superoxide generation. Values marked with different letters differed significantly (*p* < 0.05). Legends: 1—neutrophil without stimulation, 2—after stimulation LPS, 3—after stimulation PPP from L-PRP, 4—after stimulation PPP from PURE PRP, 5—after stimulation PPP from L-PRP with LPS, 6—after stimulation PPP from PURE PRP with LPS, 7—after stimulation L-PRP, 8—after stimulation PURE PRP, 9—after stimulation L-PRP with LPS, 10—after stimulation PURE PRP with LPS.

To evaluate the interactions between platelets and neutrophils we prepared co-cultures of neutrophils and platelets. Platelets were obtained from two systems for isolation of PRP, namely L-PRP and PURE PRP. Neutrophils were isolated from sheep at all studied time points, before implantation, 2 h after implantation and 24 h after implantation. All co-cultures of each PRP were incubated for 4 h and for 20 h at 37 °C and 5% CO_2_. The activity of cultures was assessed on the basis of elastase, MPO, ALP release, as well as NO and superoxide generation by neutrophils isolated from sheep in all studied time points; before implantation (Figure 3), 2 h after implantation (Figure 4) and 24 h after implantation (Figure 5). All results of cultures stimulated with L-PRP, PURE PRP, or PPP from L-PRP system and PPP from PURE PRP system (all with or without stimulation with LPS), were compared to values obtained in cultures without stimulation. Comparison between experimental and control groups was also conducted at each measurement (4 h and 20 h incubation). Our study revealed that L-PRP after addition to neutrophils culture evoked increased release of elastase in all measurements both 2 h and 24 h after implantation (marked as the experimental group) in comparison with samples without implantation (marked as the control group). At the time point 24 h after implantation (Figure 5), elastase release from the experimental group (4 h incubation) after stimulation with L-PRP rose from 53.1 ± 0.33% to 59.00 ± 0.4% (*p* < 0.05). Conversely, the value after treatment with PRP-PURE decreased to 45.00 ± 0.45%. All values in cultures treated additionally with LPS were higher. Superoxide generation in cultures of neutrophils isolated 24 h after implantation and stimulated with L-PRP generated 9.62 ± 0.2 nM of superoxide compared to a value of 5.3 ± 0.43 nM in cultures without stimulation (*p* < 0.05). Conversely, after stimulation with PURE-PRP, the obtained results were significantly lower than in L-PRP (*p* < 0.05) and similar to the unstimulated values (Figure 5). In all cultures treated with LPS, obtained values of superoxide generation were higher than in groups without addition of LPS. Additionally, as shown in Figure 3 to Figure 5, treatment of neutrophil cultures with PPP from both systems with or without addition of LPS caused decrease in neutrophil activity.

Our study revealed that, in L-PRP, previous contact of platelets with leukocytes results in increase in PMN response, whereas PURE-PRP acts as an anti-inflammatory, similarly to PPP. The responses of PMNs isolated before implantation, 2 h and 24 h after Ti implant insertion were similar. At all time points the experimental group was compared to control groups and differences between these groups were insignificant.

### 2.3. Assessment of Long-Term Interactions between Circulating Neutrophils and Ti Implants

**Figure 6:**
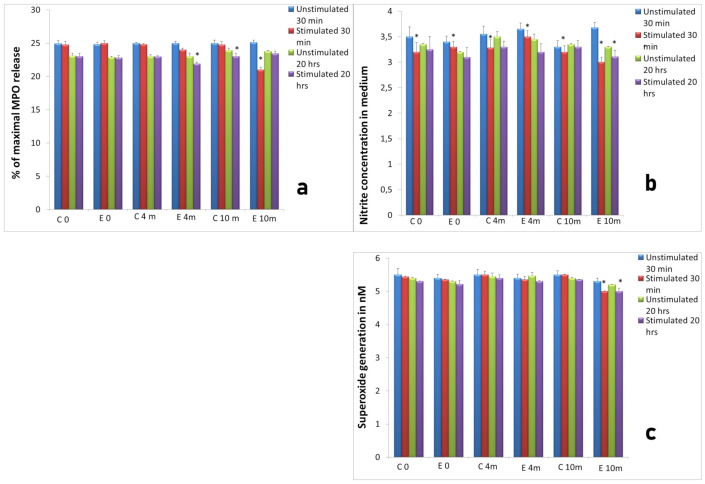
Activity of neutrophils isolated 4 and 10 months after implantation (experimental: 8 sheep) compared to control groups of neutrophils isolated at the same time points from animals from control groups (control: 4 sheep). The neutrophil response to Ti implant insertion, with or without stimulation with AMP extract; (**a**) MPO release from neutrophils, (**b**) NO generation, (**c**) superoxide generation assessed at two time points: 4 and 10 months after implantation. Two measurements, after 30 min and 20 h incubation at 37 °C and 5% CO_2_ were conducted. Data present mean values ± SE of at least three replicates for each bar. * *p* < 0.05 compared to cultures of neutrophils from the control group at the same time points. Legends: C0—control group before surgery, E0—experimental group before surgery, C 4m—control group 4 months after, E 4m—experimental group 4 months after, C 10m—control group 10 months after, E 10m—experimental group 10 months after insertion of a titanium implant.

The response of circulating neutrophils to implantation was examined 4 months and 10 months after insertion of Ti implant into the ovine tibia and compared to control group. We did not observe significant differences between results obtained in experimental group and control group of sheep with respect to neutrophil activity at both time points. Similarly, as in the first part of the experiment, the treatment of neutrophil cultures with AMP extract for 30 min and 20 h evoked decreased release of MPO, nitrite concentration and superoxide generation in comparison to unstimulated cells (Figure 6a–c).

As shown in Figure 6b the concentration of nitrite in medium significantly (*p* < 0.05) decreased after stimulation of neutrophils with AMP extract in measurements 4 and 10 months after implantation compared to the control (Figure 6b). Superoxide generation decreased significantly (*p* < 0.05) in cultures obtained 10 months after implantation (Figure 6c).

**Figure 7:**
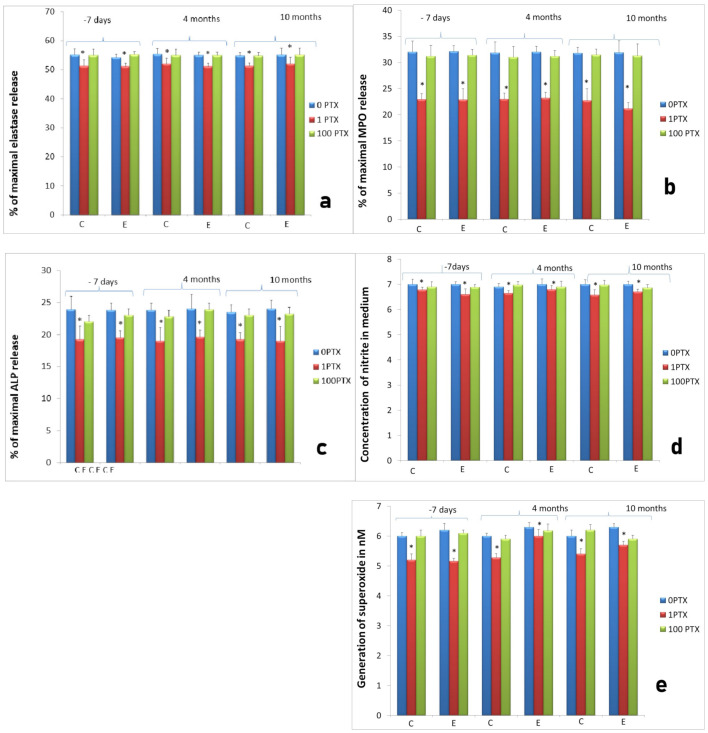
Activity of neutrophils isolated 4 and 10 months after implantation (experimental—8 sheep) compared to control groups of neutrophils isolated at the same time points from animals from control groups (control: 4 sheep). C—control group, E—experimental group. The response to implantation and treatment with two concentrations of PTX (1 and 100 µg/mL); (**a**) elastase release from neutrophils, (**b**) MPO release, (**c**) ALP release, (**d**) NO generation, (**e**) superoxide generation assessed at two time points: 4 and 10 months after implantation. Data present mean values ± SE of at least three replicates for each bar. * *p* < 0.05 compared to cultures of neutrophils without stimulation (0 µg/mL) at the same time points.

The effect of PTX on neutrophil response was concentration-dependent, as in the previous experiment on the inflammatory phase of the repair process. After stimulation with 1 µg/mL the significant decrease in elastase, MPO and ALP release as well as NO and superoxide generation was noted, whereas at a concentration of 100 µg/mL the changes were insignificant in cultures 4 months after implantation. Measurements repeated after 10 months gave the similar effect depicted in Figure 7.

**Figure 8:**
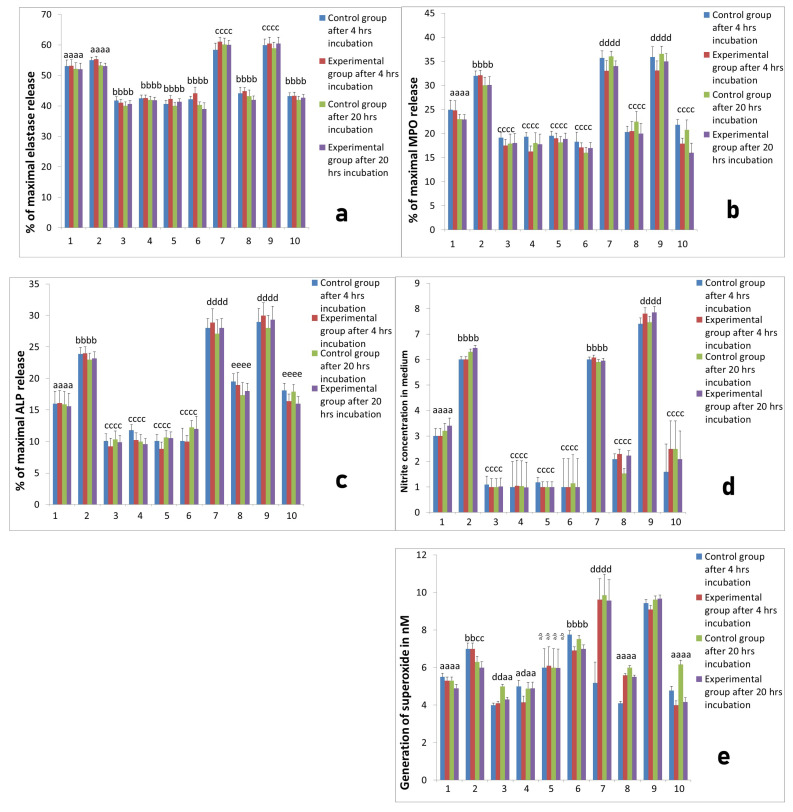
The response of neutrophils isolated 4 months after implantation to different PRP-derived products with or without additional stimulation with LPS. Cultures were incubated for 4 h or for 20 h at 37 °C and 5% CO_2._ Data present mean values ± SE of at least three replicates for each bar. The neutrophil response was assessed with respect to: (**a**) elastase release from neutrophils, (**b**) MPO release, (**c**) ALP release, (**d**) NO generation, (**e**)-superoxide generation. Values marked with different letters differed significantly (*p* < 0.05). Legends: 1—neutrophil without stimulation, 2—after stimulation LPS, 3—after stimulation PPP from L-PRP, 4—after stimulation PPP from PURE PRP, 5—after stimulation PPP from L-PRP with LPS, 6—after stimulation PPP from PURE PRP with LPS, 7—after stimulation L-PRP, 8—after stimulation PURE PRP, 9—after stimulation L-PRP with LPS, 10—after stimulation PURE PRP with LPS.

**Figure 9:**
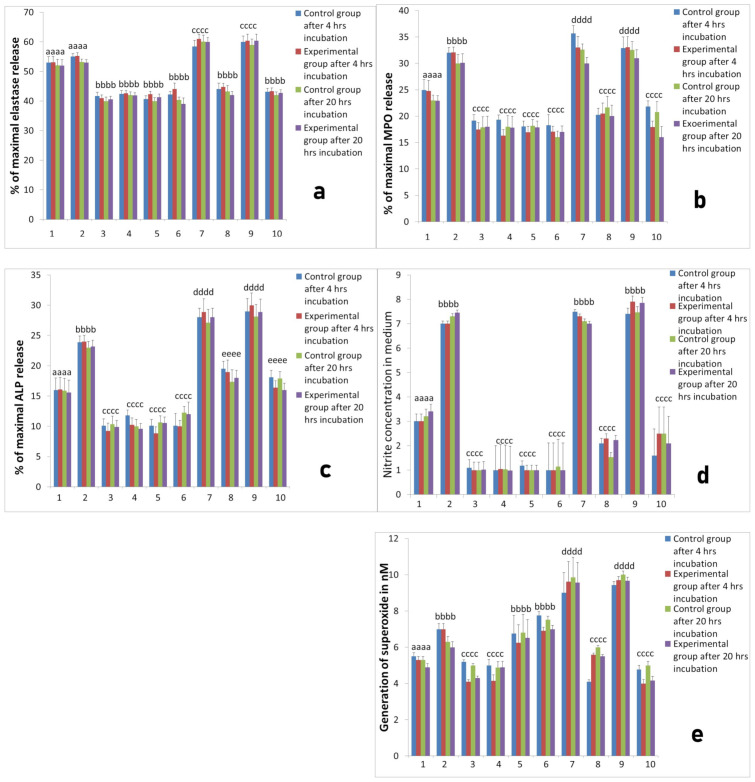
The response of neutrophils isolated 10 months after implantation to different PRP-derived products with or without additional stimulation with LPS. Cultures were incubated for 4 h or for 20 h at 37 °C and 5% CO_2_. Data present mean values ± SE of at least three replicates for each bar. The neutrophil response was assessed with respect to: (**a**) elastase release from neutrophils, (**b**) MPO release, (**c**) ALP release, (**d**) NO generation, (**e**) superoxide generation. Values marked with different letters differed significantly (*p* < 0.05). Legends: 1—neutrophil without stimulation, 2—after stimulation LPS, 3—after stimulation PPP from L-PRP, 4—after stimulation PPP from PURE PRP, 5—after stimulation PPP from L-PRP with LPS, 6—after stimulation PPP from PURE PRP with LPS, 7—after stimulation L-PRP, 8—after stimulation PURE PRP, 9—after stimulation L—PRP with LPS, 10—after stimulation PURE PRP with LPS.

**Figure 10:**
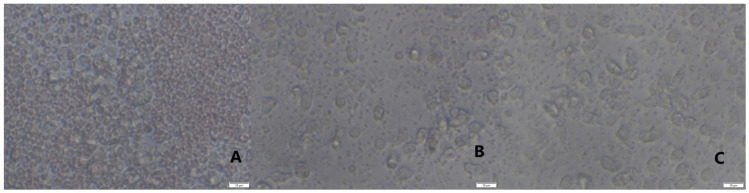
Representative phase contrast microscopical images. (**A**) Isolated neutrophils, (**B**) co-culture of activated PMNs with platelets after addition of PURE PRP, (**C**) co-culture of activated PMNs with platelets after addition of L-PRP. Original magnification × 40 (Olympus-CK40).

L-PRP after addition to neutrophils culture evoked increased release of elastase in all measurements both 4 months (Figure 8a) and 10 months (Figure 9a) after implantation in comparison with cultures of unstimulated neutrophils. After stimulation with LPS, higher response of the neutrophil/platelets co-cultures was observed. Treatment with PURE-PRP, in turn, caused diminished elastase release at both time points after 4 months (Figure 8a) and after 10 months (Figure 9a). In the case of the other studied PMNs parameters, namely enzymatic response and reactive oxygen and nitrogen species (RONS) generation, we noted increased response to addition of L-PRP. All values were higher after stimulation of cultures with LPS (Figure 8 and Figure 9). Contrary to this, PURE-PRP caused a decrease in these activities. Morphological assay of neutrophil/platelet coculture indicated the presence of activated PMNs both in L-PRP and PURE-PRP compared to isolated PMNs without platelets (Figure 10A–C).

## 3. Discussion

Our study revealed that Ti implant insertion into the ovine tibia did not evoke any side effects neither at the inflammatory phase of repair process nor at long-time treatment 4 and 10 months after surgery. According to some authors, implantation of biomaterial elicits an immune response that causes tissue damage and activation of neutrophils which initiate a cascade of inflammatory events [24]. However, the interactions between neutrophils and implanted biomaterials varied depending on many factors, especially the material used for implantation. Previously, Kumazawa et al. [6] assessed neutrophil functions after contact of neutrophils with Ti particles for 1 month at 37 °C. Their study revealed different response depending on Ti particles size, namely fine Ti particles below 10 μm in diameter induced cytotoxicity both in vivo and in vitro, whereas large particles, as used in implants, are biocompatible. Abaricia et al. analyzed in vitro response of neutrophils to different surfaces of the Ti discs [22]. These authors confirmed that the biomaterial surface modulates cell activation. Neutrophils were differentially activated after contact with smooth, rough, or rough–hydro Ti surfaces and different secretion of inflammatory molecules, enzymes, and formation of NETs was observed. Additionally, they show that activated neutrophils are key cells for macrophage polarization [22]. In our study, circulating neutrophils were not activated neither at the inflammatory phase nor after long term evaluation of host/Ti implants interactions. These findings were confirmed with a lack of systemic inflammatory response on the basis of hematological results and APP response. To our knowledge, this is a first study on the first inflammatory phase and long-term interactions between circulating neutrophils and Ti implant on ovine model of surgical procedures.

We estimated that addition of autologous AMP extract caused a decrease in neutrophil in vitro activity to a different extend, enzymatic activity of primary granules of cultured neutrophils decreased insignificantly, whereas enzyme release from secondary granules and RONS generation were significantly diminished. Previous findings indicated that autologous rabbit AMP extract decreased neutrophils and macrophages activity during osteochondral graft implantation [12], whereas heterologous (porcine) AMP extract stimulated ovine macrophages to increase pro-inflammatory response after implantation of a Ti implant [11]. Moreover, Huiwen et al. indicated that some AMPs, apart from well known antimicrobial activity, also exert a potent anti-inflammatory effect [25]. Our results show that an autologous ovine AMP extract did not cause strong inhibition of PMNs’ activity, it rather modulated them towards diminished response. Other authors demonstrated the role of short natural peptides in enhancement of neurogenic differentiation capacity in vitro [26]; this research paves the way for clinical studies on the application of natural peptides in regenerative medicine.

We observed that PTX in a concentration of 1 µg/mL significantly decreased enzyme release and RONS generation in measurements before and after Ti plate implantation in an ovine model. Some previous studies indicated the potential of PTX as immunomodulatory and anti-inflammatory factor, that suppresses production of TNF by human and murine leukocytes as well as superoxide production by neutrophils and their degranulation [27]. Previous studies about the therapeutic potential of PTX in regenerative medicine are contradictory. Some authors did not confirm the influence of PTX on histological changes during the tissue repair process [28]; conversely, others stated that PTX improved bone healing in rats [14]. Additionally, Chalmeth described the effectiveness of the anti-inflammatory effect of PTX on sheep and underlined its therapeutic potential in serious inflammatory disorders [29].

We found that different neutrophil responses to platelets depended on the PRP system used for preparation of platelet concentrates. L-PRP activated neutrophils, whereas PRP PURE decreased their enzymatic response and RONS generation. In our experiment, after addition of L-PRP, we observed increased enzyme release as well as production of NO and superoxide in these co-cultures, especially after stimulation with LPS. It is known that platelets are important regulators of inflammation, they express Toll-like receptors, and can enhance leukocyte effector functions including pro-inflammatory activity [9]. Previously, some authors have shown that PRP reach in leukocytes stimulated some functions of neutrophils and activated platelets, contributing to pro-inflammatory activities [8,30]. Gros et al. underlined the role of activated platelets in regulation of neutrophil functions such as the ROS generation, the secretion of neutrophil granule content, phagocytosis, or the formation of neutrophil extracellular traps [30]. We supposed that the increased activity of neutrophils in co-cultures with platelets from L-PRP resulted from previous crosstalk with leukocytes present in this platelet concentrate. This statement could be confirmed by observation that platelets derived from PRP PURE without leukocytes caused decreased inflammatory response in neutrophils in co-culture, as estimated on decreased enzyme release and RONS generation, both with or without stimulation with LPS. Additionally, some authors [15] noted that neutrophil activation is reduced in the presence of platelets. Anti-inflammatory potential of the interactions of platelets and neutrophils was also found by Lana et al. [17]. They estimated that the combination of neutrophils with platelets could have a more beneficial than detrimental effect on tissue repair process. Activated platelets release arachidonic acid which is captured by neutrophils and converted into leukotriene and prostaglandins. Alternatively, platelets in association with neutrophils can pick up leukotrienes and covert them into lipoxin, a potent anti-inflammatory protein capable of limiting neutrophil activation and preventing diapedesis, thereby promoting the resolution phase of the healing cascade. Thus, these interactions seem to be beneficial for resolution of inflammation.

We observed the inhibitory effect of PURE PRP on neutrophil enzymatic response in all the studied time points. Additionally, other authors found that platelets can inhibit the release of elastase and MPO from neutrophils [31]. The anti-inflammatory effect of PRP seems to rely in different mechanisms and could dampen elastase secretion as a mechanism to prevent inflammatory host damage [15]. It is possible that tromboxane (TXA) from activated platelets affects the oxygen metabolism of neutrophils, which lack this enzyme. This indicated the significant influence of platelets on neutrophils’ function [32]. However, it should be taken into account that the pro- or anti-inflammatory effect of platelets concentrate depends on experimental conditions such as the platelet and neutrophil stimulators and other factors [30]. Thus, it appeared that platelets regulate neutrophil degranulation and ROS production in a stimulus-specific manner and many different conditions should be taken into account. It appeared that the pro- or anti-inflammatory effect of platelets is highly dependent on such factors as the cause of inflammation, which defines the pathways and extent of leukocytes’, platelets’, and endothelial cells’ activation [30]. Hence, the differences in neutrophil response depend on the PRP preparation used.

It should be underlined that the overall impact of platelets on inflammatory process can either be beneficial or deleterious depending on the pathophysiological context [30]. Some authors stressed the key role of PRP in inflammatory aspects of tissue healing involved in release of major anti-inflammatory growth factors, as shown in the experiment on PRP treatment of tendon cells in vitro described by Zhou and Wang [21]. PRP could also decrease the release of pro-inflammatory cytokines. However, leukocytes in PRP could potentially enhance inflammation because they significantly increased the gene and protein expression of IL-1*β*, IL-6, and TNF-*α* in tendon cells. This demonstrates that leukocytes can exacerbate inflammation in tendon cells but pure PRP without leukocytes can be anti-inflammatory [21]. Generally, different methods of action of different platelet concentrates are useful in different applications in regenerative medicine. Exacerbated tissue inflammatory levels can be necessary in chronic wound treatments. However, neutrophils can also cause tissue damage and, thus, should not be used in some applications. Zhou and Wang [21] found that the PRP used together with neutrophils may change the collagen type III/type I ratio, leading to fibrosis and decreased tendon strength.

Our study revealed that, depending on the system used for isolation of platelets, we could obtain different effects of PRP concentrates on neutrophil response and different pro- or anti-inflammatory responses of cultured cells. The stimulation with LPS generated higher values in all studied groups. The immune functions of platelets are various and diverse and depend on the combination of different stimuli received by the neutrophil/platelet populations. These responses can cause either augmentation or attenuation of neutrophil functions both in the absence and presence of TLR stimulation with LPS [9]. The inflammatory phase is necessary for healing process, especially the remodeling and tissue contraction phase [17]. To summarize, platelet–leukocyte interactions can regulate inflammation, wound healing and tissue repair. TLR-4 on platelets stimulates platelet–neutrophil interactions and modulates neutrophil response [33].

In our study, addition of PPP±LPS into neutrophil cultures caused decrease in their activity both in comparison with unstimulated cultures and in comparison with cultures stimulated with both systems of PRP preparation. Some active biological factors, both anabolic and catabolic were found in PPP and could be used in regenerative medicine, especially in treatment of some musculoskeletal disorders involved in exacerbated inflammatory response [34]. The anti-inflammatory potential of AMP extract, PTX, and PURE PRP could be used to prevent excessive inflammation, which could lead to aseptic implant loosening and graft rejection. Otherwise, pro-inflammatory action could be applied in treatment of chronic conditions and in defective inflammatory response to enhance healing.

Generally, it is a concern that therapeutic inhibition of neutrophil activity, because of their antimicrobial role, will trigger infections. However, some reports suggest the therapeutic window of attenuation neutrophil-mediated inflammatory process without interfering with host defense depended on neutrophils response [35]. Our research described possible modifications of neutrophil activity and broadens understanding of interactions neutrophils/biomaterial beyond the required biocompatibility assays, enabling further reduction in side effects involved in implantation of biomaterials. This is only preliminary study on limited number of animals. Evaluated compounds before introducing into clinical practice will need careful clinical trials. It should be also taken into account that in patients all interactions organism/biomaterial could additionally affect the studied response. However, the results obtained during in vitro studies constitute a promising basis for further research.

## 4. Materials and Methods

### 4.1. Animal Model and Surgical Procedure

**Figure 11:**
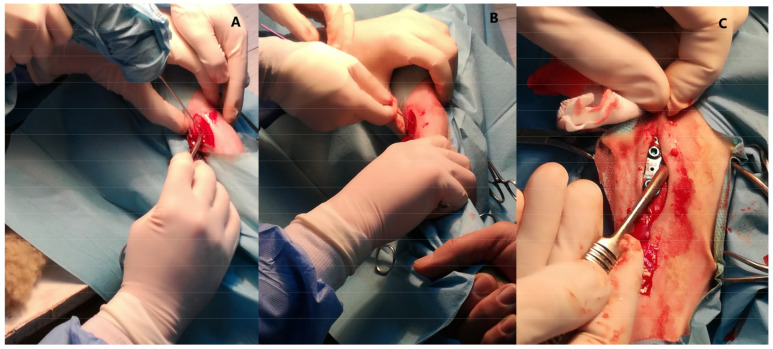
A clinical photographs of implant insertion on the ovine tibia: (**A**) dissection of soft tissue and (**B**) periosteum. (**C**) implants installed in the right tibia.

The study was conducted in a group of 12 healthy (based on clinical examination and blood analysis) female sheep (*Ovis aries*, BCP local breed), aged 4 months (weight about 20 kg), from the Bezek Experimental Farm of University of Life Sciences in Lublin (Poland). The animals were randomly divided into two groups, a control group (*n* = 4) and an experimental group (*n* = 8). All animals were fed, housed and cared for in accordance with the directive concerning the protection of animals. The experiment was approved by the local Ethics Committee number II in Lublin (No 84/2019). All animals from the experimental group had a Ti implant inserted into the tibia (Figure 11A–C).

The sheep were premedicated with intramuscular injection of xylazine (0.1 mg/kg) and Butorphanol (0.1 mg/kg). The surgical area was aseptically cleaned, and the standard surgical approach for proximal tibia was prepared then, after periosteal elevation, a Ti eight-plate implant with two screws was implanted. Postoperative medication included Melovem (Meloxicam 5%, Dopharma Research B.V. 1.2 mL SC) as analgesic and Combi-ject (200 000 IU/mL Penicillin and 200 mg/mL Streptomycin) to prevent infection. All animals were monitored postoperatively for ten days in terms of the condition of the animal, assessment of breathing, heart rate, and body temperature. Additionally, monitoring of the area of the postoperative wound and examination of the skeletal system to exclude motor disorders of the operated limb was performed.

Peripheral blood for preparation of AMP extract and for hematological assays was drawn from the jugular vein seven days before the experimental procedure.

Hematological parameters in all sheep were within the reference ranges, as estimated after blood count analysis using an Abacus Junior Vet analyzer (Diatron, Budapest, Hungary). Acute phase response on the basis of two indicative proteins namely haptoglobin and fibrinogen concentration in plasma were conducted as described earlier [36].

AMP extract was prepared as previously described [37]. Briefly, after isolation from whole blood, neutrophils were centrifuged and homogenized to obtain neutrophil granules. Isolated granules were then stirred in 10% acetic acid at 4 °C overnight. The solution of AMP was lyophilized and stored at −70 °C. The peptide content was assessed spectrophotometrically on the basis of absorbance of Tyr at 280 nm using an extinction coefficient of 1450 M^−1^ cm^−1^ [38].

### 4.2. Neutrophils Isolation, Culture and Stimulation

**Figure 12:**
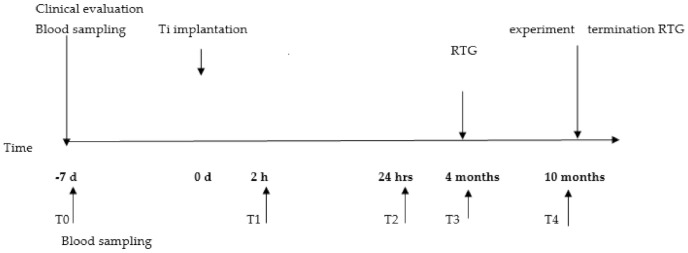
Timeline for clinical evaluation, surgery and blood sampling.

For evaluation of the circulating neutrophils during the inflammatory phase response, blood samples were collected into EDTA tubes at the following time points: first T0: 7 days before surgery, second T1: 2 h after surgery and last T2: 24 h after surgery. For evaluation of long-term neutrophil/implant interactions, blood was also drawn 4 months (T3) and 10 months (T4) after implantation. Blood from control sheep was obtained at the same time points (Figure 12).

Once neutrophils had been isolated from the blood, the number and viability of the obtained cells were determined using an R1 Automated Cell Counter (Olympus, Warsaw, Poland) and then the cells (98% viable) were plated at a density of 2.0 × 10^6^ cells/mL. The neutrophils were 85% pure, as under staining with Giemsa–May Grunewald, the contamination of platelets was less than 1 platelet per 10 neutrophils. Then, the cell suspensions were supplemented as follows: the control group with phosphate-buffered saline (PBS) (marked as unstimulated) and the stimulated group with AMP at a concentration of 20 μg/mL (marked as stimulated). Then, both stimulated and unstimulated cultures were incubated for 30 min and for 22 h at 37 °C in the presence of 5% CO_2_. Other groups were treated with different concentrations of pentoxifylline (Polfillin Polpharma Poland) added to final concentrations of 0, 1, and 100 µg/mL of culture and incubated for one hour at 37 °C in the presence of 5% CO_2_.

### 4.3. Platelet-Rich and Platelet-Poor Products

Platelet-rich plasma and platelet-poor plasma were isolated using two commercial systems. System 1 (CURASAN) was used to prepare L-PRP. Blood samples of 8.5 mL were collected from the jugular vein into a monovette containing citrate phosphate, dextrose, and adenosine (CPDA) from a PRP kit (Curasan AG, Kleinostheim, Germany). A two-step procedure according to the manufacturer’s instructions was applied. The top layer (platelet-poor plasma (PPP)) was collected; the remaining volume was platelet-rich plasma (PRP) [36]. The second system was a XERTHRA PRP kit used to prepare PURE PRP and PPP according to the manufacturer’s instructions.

The cell content in both systems was evaluated with a hematological analyzer. Hematological evaluation confirmed data provided by the manufacturers of the PRP systems. The platelet number was determined using a Bȕrker chamber. Morphological studies showed discoid, solitary platelets displaying no signs of activation due to the preparation procedure. PRP was adjusted to 2.5 × 10^8^ platelets/mL in phosphate-buffered saline (PBS). PPP was diluted in a similar volume of PBS.

#### Neutrophil/Platelets Coculture

Isolated neutrophils were cultured with autologous platelets obtained from one of two systems in a ratio of 1:250 neutrophils:platelets. An equal amount of PPP was added to neutrophil cultures; cultures of neutrophils without other platelet related products were used as control. All cultures of neutrophils ± platelets obtained from each of the two systems were left unstimulated or were stimulated with 1 ng/mL lipopolysaccharide (LPS) from *E. coli* serotype 055:B5 (Sigma-Aldrich, Poland) as a TLR4 agonist. The cultures were incubated for 4 h and for 20 h at 37 °C and 5% CO_2_ [9].

### 4.4. Evaluation of Cells and their Function

The studied cultures’ neutrophils (1 × 10^6^/mL) ± platelets were incubated at 37 °C in 5% CO_2_ in 96-well plates for 4 h in the presence of buffer or LPS. Then, neutrophil ± platelets cultures were subjected to microscopic analysis of the morphology using an inverted microscope (Olympus) at 40 × magnification.

For functional analysis, enzyme release from azurophilic granules was assayed on the basis of elastase and myeloperoxidase (MPO) release and compared to the maximum enzyme content obtained after the treatment of the cells with 0.5% Triton X-100 (Sigma–Aldrich, Poznan, Poland). The elastase activity was determined using a specrophotometric method with a substrate azocasein (Sigma-Aldrich). Myeloperoxidase (MPO) release was determined by measuring the absorbance at 490 nm after 10 min incubation of the sample with an equal volume of o-phenylendiamine (Sigma-Aldrich, Poznan, Poland). A marker of specific granule response, alkaline phosphatase (ALP) activity was measured at 405 nm after 10 min incubation at 25 °C with 4-nitrophenyl phosphate disodium salt hexahydrate (Sigma-Aldrich, Poznan, Poland).

Nitric oxide (NO) level was determined by means of the Griess reaction [36]. Briefly, equal volumes of the culture supernatant and Griess reagent (0.1% N-[1-naphtyl] ethylendiamine dihydrochloride 1% sulphanilamide and 2.5% H_3_PO_4_) were mixed and incubated at room temperature for 10 min and absorbance was measured. The values obtained were expressed as nitrite concentration (a stable breakdown product of NO, which accumulates in the medium). Absorbance was converted into micromoles (µM) based on a NaNO_2_ standard curve. Superoxide anion (O_2_^−^) production per 10^6^ neutrophils was assayed after incubating neutrophils with 0.1% nitroblue tetrazolium solution (Sigma-Aldrich, Poznan, Poland) at room temperature for 10 min by reading absorbance at 545 nm. Generation of superoxide was assessed using the extinction coefficient 21.1 nM.

Measurements were performed as follows: for estimation of the influence of AMP on neutrophils, after 30 min and 20 h incubation; for assessment of PTX influence on neutrophils, after 1 h; and in neutrophil/platelets co-cultures, after 4 h and 20 h incubation in 37 °C at 5% CO_2_.

### 4.5. Statistical Analysis

Statistical analysis was performed using Statistica 13.1 Software (StatSoft, Poland). The experimental results were expressed as the mean ± standard error (SE). Multiple-group comparisons were made using either Student’s t test or one-way ANOVA analysis. *p* values less than 0.05 were considered significant.

## 5. Conclusions

Over the course of this long-term evaluation of interactions between circulating neutrophils and an implanted Ti plate, we estimated that neither in the inflammatory phase of the repair process nor during next 10 months was deleterious neutrophil activity seen. Moreover, we did not detect any adverse effects at the implant site or systemic inflammatory response. We also evaluated some products as potential modifiers of neutrophils and inflammatory response. Our study revealed that PTX, AMP extract, PURE PRP and PPP acted as anti-inflammatories and decreased neutrophil response, whereas L-PRP enhanced this response, thus acting as a pro-inflammatory. These findings are applicable in modulation of inflammatory response during implantation of biomaterial and create the possibility of modulating the cells response towards pro- or anti-inflammatory actions in order to reduce host tissue damage. Simultaneously, these results broaden our knowledge about biomaterial–host interactions.

## Figures and Tables

**Table 1:** Mean hematological and APP parameters (±SE) in sheep (*n* = 8) before (T0), after 24 h (T2), after 4 months (T3) and after 10 months (T4) surgery.

Parameter	T0	T2	T3	T4
WBC (10^9^/L)	5.99 ± 1.22	5.9 ± 1.9	6.13 ± 1.46	5.95 ± 1.34
GRAN (10^9^/L)	3.4 ± 1.1	3.35 ± 0.97	3.67 ± 1.46	3.42 ± 0.98
RBC (10^12^/L)	9.2 ± 0.91	9.06 ± 0.82	9.1 ± 0.79	9.15 ± 0.9
HCT (%)	27.45 ± 3.21	27.12 ± 2.45	27.57 ± 2.32	27.34 ± 3.11
HGB (g/L)	113.2 ± 1.22	117.9 ± 1.14	114.8 ± 1.03	117.5 ± 1.06
Fibrinogen (g/L)	4.10 ± 1.00	4.97 ± 0.51	4.52 ± 0.62	4.15 ± 0.86
Haptoglobin (g/L)	0.22 ± 0.20	0.61 ± 0.13	0.21 ± 0.15	0.28 ± 0.22

## Data Availability

The data presented in this study are available on request from the corresponding author.
